# Novel biallelic *CDK9* variants are associated with retinal dystrophy without CHARGE-like malformation syndrome

**DOI:** 10.1038/s10038-025-01395-1

**Published:** 2025-09-16

**Authors:** Sachiko Nishina, Kaoruko Torii, Shizuka Ishitani, Tomoyo Yoshida, Maki Fukami, Kenji Kurosawa, Kenjiro Kosaki, Hirotomo Saitsu, Tohru Ishitani, Yoshihiro Hotta

**Affiliations:** 1 Division of Ophthalmology, National Center for Child Health and Development, Tokyo, Japan; 2 Department of Ophthalmology, Hamamatsu University School of Medicine, Shizuoka, Japan; 3 Department of Homeostatic Regulation, Research Institute for Microbial Diseases, Osaka University, Osaka, Japan; 4 Department of Molecular Endocrinology, National Research Institute for Child Health and Development, Tokyo, Japan; 5 Center for Medical Genetics, National Center for Child Health and Development, Tokyo, Japan; 6 Center for Medical Genetics, Keio University School of Medicine, Tokyo, Japan; 7 Department of Biochemistry, Hamamatsu University School of Medicine, Shizuoka, Japan

**Keywords:** Medical genetics, Developmental biology

## Abstract

Cyclin-dependent kinase 9 (CDK9) phosphorylates the *C*-terminal domain of RNA polymerase II (RNAPII) to regulate transcription. Previously, we reported that an 8-year-old boy with the biallelic *CDK9* variants p.A288T and p.R303C exhibited a CHARGE-like malformation syndrome in which retinal dystrophy was a distinguishing feature. This dystrophy was caused by the decreased CDK9 kinase activity associated with these variant alleles [wild-type (WT) > A288T > R303C]. In this study, we describe a female patient who also bears biallelic *CDK9* variants but displays retinal dystrophy without a CHARGE-like malformation syndrome. Trio-based whole-exome sequencing identified a new variant *CDK9* allele, p.P321S, that occurred de novo in the patient. As a result, this female patient displayed compound heterozygous variants composed of the p.A288T *CDK9* variant of maternal origin plus the novel p.P321S variant. With respect to reduced kinase activity, the new variant could be ranked as WT > P321S > A288T. Thus, our study raises a possibility that retinal dystrophy can arise with or without a CHARGE-like malformation syndrome depending on the level of kinase activity associated with the combination of variant *CDK9* alleles present.

## Introduction

Cyclin-dependent kinase 9 (CDK9) binds to cyclin T1 to form the positive transcription elongation factor b complex, which regulates transcription [1]. A major target of CDK9 kinase activity is the C-terminal domain of RNA polymerase II. Because of its essential role in numerous cellular responses, CDK9 is evolutionarily well conserved from yeast to zebrafish to humans. In the context of human disease, hyperactivation of CDK9 leads to the development of various cancers, including acute myeloid leukemia. CDK9 has thus been deemed an attractive target for new cancer therapies. However, the precise functions of CDK9 in human physiological processes such as morphogenesis are still not fully understood. Filling such gaps in our knowledge of CDK9 biology is essential for the rational design of new treatments that will not impose harmful side-effects.

CHARGE syndrome (CS) is a congenital anomaly syndrome caused by a variant in the *CHD7* gene, which is necessary for fetal development. A deficiency in CHD7 function results in delayed fetal growth, audiovisual disturbances, and multi-system internal organ disease [2]. Several genetic studies have reported on families with children who had CHARGE syndrome-like features but normal *CHD7* genes. For example, a pair of cousins from a single consanguineous family showed CHARGE syndrome-like features, including coloboma, renal malformation, restricted growth, and limb anomalies, but expressed a rare variant of the *CDK9* gene, namely p.R225C [3]. In addition, three patients with CHARGE syndrome-like features from three different families were homozygous for this same *CDK9* variant [4]. As part of the “Finding of Rare Disease Genes” (FORGE) Strategies for Gene Discovery initiative [5], we also previously reported on a patient who was compound heterozygous for two rare pathogenic *CDK9* variants, namely p.A288T and R303C [6]. Thus, biallelic deficiency of CDK9 activity is now an established cause of a human multiple malformation syndrome that involves the eyes and features vision-threatening retinal dystrophy. In the present study, we report the identification of a different compound heterozygous individual with the biallelic *CDK9* variants p.A288T/P321S. Interestingly, this patient displayed retinal dystrophy without the CHARGE-like multiple malformation syndrome. Thus, our study raises a possibility that phenotypic spectrum of biallelic *CDK9* variants may extend to retinal dystrophy without CHARGE-like features.

## Case report

### Clinical description

The proband was an 11-year-old girl who was born at 38 weeks gestational age to healthy nonconsanguineous parents and weighed 2724 g. At the age of one year, she was referred to the Division of Ophthalmology, National Center for Child Health and Development (NCCHD) for a thorough examination of left eye strabismus and bilateral ptosis. She also had a history of infant gastrointestinal allergy. Her parents and elder brother had no notable diseases.

**Fig. 1:**
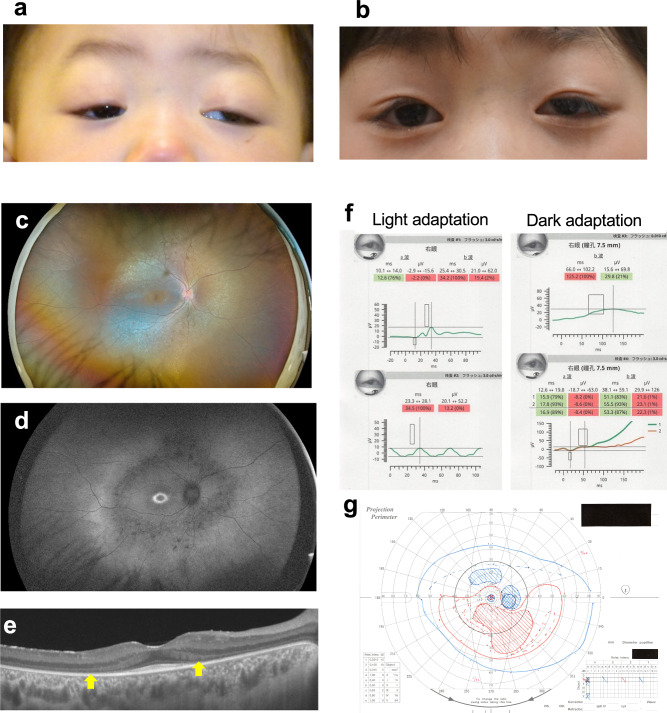
The ophthalmic phenotypes of the patient. **a** Photograph showing the bilateral ptosis, and left exotropia and hypertopia, seen in the patient at the age of 14 months. **b** Photograph showing orthotropia in the patient at the age of five years following surgical treatment to correct her bilateral ptosis and left exotropia. **c** Ultra-widefield fundus photograph showing extensive retinal degeneration with ossicle-like pigmentation in the mid-periphery in the patient at the age of 11 years. **d** Fundus autofluorescence photograph demonstrating a hyperfluorescent ring around the fovea, and hypofluorescence in the mid-periphery where retinal degeneration was prominent, in the patient at the age of 11 years. **e** Swept-source optical coherence tomography image of the posterior retina showing severe thinning of the outer nuclear layer and a diminished ellipsoid zone (arrows) except in the foveal area. The patient was 11 years old. **f** Full-field electroretinography using the RETeval^TM^ portable device revealing reduced cone responses (LA 3.0 and 30 Hz flicker), and markedly decreased rod responses (DA 0.01) and mixed rod and cone responses (DA 3.0), in the patient at the age of 11 years. **g** Goldman perimetry showing constricted visual fields with large paracentric scotomas in the patient at the age of 11 years

The initial ocular examination of the patient at the age of one year revealed congenital bilateral ptosis and left exotropia and hypertropia due to dissociated vertical deviation (Fig. 1a), but she maintained binocular fixation by elevating her chin. There were no abnormal findings in either the anterior segment or the fundus of either eye. No specific neurological or systemic abnormalities were identified. She underwent strabismus surgery for left exotropia at 2 years and 9 months of age and frontalis suspension for bilateral ptosis at 4 years and 9 months of age. At the age of 5 years, she achieved orthotropia (Fig. 1b) with coarse stereoacuity of 3000 s of arc on the Stereo Fly Test. Thereafter, periodic examinations showed no significant changes. However, at 8 years of age, ophthalmoscopy showed bilateral degeneration of the retinas necessitating comprehensive and detailed ophthalmic examinations. Around age 9 years, the patient’s night blindness and photophobia became significant. Ultra-wide fundus photography and autofluorescence imaging (California, Optos Plc, Dunfermline, UK) revealed extensive retinal degeneration and hypofluorescence in the mid-periphery at age 11 years (Fig. 1c, d). Central retinal architecture was evaluated using swept-source optical coherence tomography (SS-OCT) (DRI OCT-1, Topcon, Tokyo, Japan), which showed bilateral significant thinning of the ONL and a diminution of the EZ except for the foveal area (Fig.1e). Using ffERG employing the RETeval system (LKC Technologies, Inc. Gaithersburg, MD, USA), we observed decreased cone responses and markedly reduced rod responses in both eyes (Fig.1f). These findings confirmed a definitive diagnosis of bilateral rod-cone retinal dystrophy. The best-corrected decimal visual acuity was 0.7 in the right eye and 0.5 in the left eye. Goldman perimetry showed constricted visual fields with large paracentric scotomas in both eyes (Fig.1g). Systemic re-evaluations revealed mild developmental delay and slightly shorter-than-normal stature, but there were no other CHARGE syndrome-like features; neither atresia of the choanae, heart defects, ear abnormalities, hearing loss, genital anomalies, nor the hockey stick sign were present. The patient’s mother had no ocular or systemic abnormalities, although she did prove to be a carrier of one of the *CDK9* variants.

### Molecular studies

**Fig. 2:**
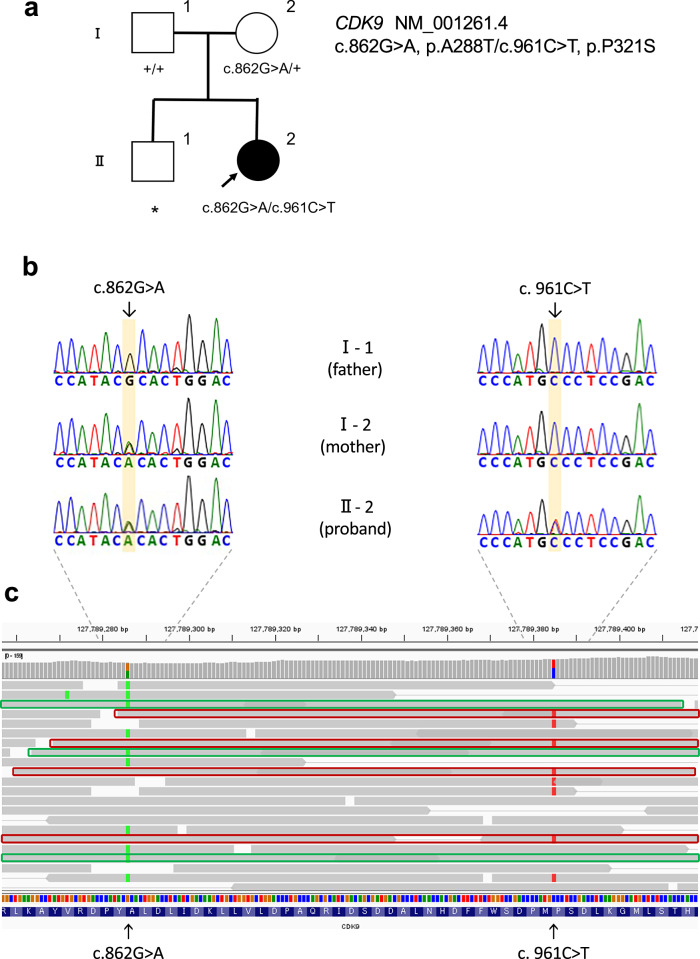
A novel CDK9 genetic variant in the patient. **a** Pedigree of the patient’s family showing *CDK9* variants. The *CDK9* genotypes of the proband (II-2) and parents (I-1 and I-2) are shown. Arrow, proband; square, male; circle, female; black, affected individual; +, wild-type allele; *, DNA sample not available. **b** Electropherograms of *CDK9* variants in the proband and parents. Partial *CDK9* sequences from the proband (II-2) and parents (I-1, I-2) are shown. Variant positions are highlighted in yellow. The c.862G>A variant is maternally inherited, whereas the c.961C>T variant is absent in both parents, indicating a de novo origin in the patient. **c** IGV view of *CDK9* variants in the proband showing that the c.862G>A and c.961C>T variants are located on different alleles (in trans). The c.862G>A variant is highlighted in light green and the c.961C>T variant in red. The allele carrying c.862G>A is outlined in green, and the allele carrying c.961C>T is outlined in red

To determine the genetic basis of the patient’s eye anomalies, genomic DNA was extracted from peripheral blood samples of the patient and her parents. Trio-based whole-exome sequencing was performed. Exome capture was carried out using the Twist Exome 2.0 (Twist Bioscience, South San Francisco, CA, USA), and sequencing was performed on a NovaSeq 6000 (Illumina, San Diego, CA, USA) with 150-bp paired-end reads. Exome data processing, variant calling, and variant annotation were performed as described previously [7]. Using PLINK (v1.90; https://www.cog-genomics.org/plink/1.9/) [8], we calculated PI_HAT based on 4,225 variants from the trio samples together with 359 individuals from the 1000 Genomes Project. The PI_HAT values between the patient and each parent were 0.5 (Z1 = 1), thereby confirming both paternity and maternity. Read-depth-based copy number variation analyses were performed using the exome-hidden Markov model (XHMM) [9] and jNord [10]. After the filtering steps, we identified two rare missense variants (c.862G>A, p.A288T and c.961C>T, p.P321S) in the *CDK9* (NM_001261.4) gene. The c.862G>A variant was maternally inherited, while c.961C>T was not found in either parent (Fig. 2a, b). Visualization with the Integrative Genomics Viewer (IGV) confirmed that c.862G>A and c.961C>T were present on different reads, indicating that they are in trans (Fig. 2c). The c.862G>A variant was previously reported by us as a causative variant in CHARGE syndrome-like retinal dystrophy [6]. The c.961C>T variant was considered to be a novel de novo variant that was absent from population allele frequency databases such as gnomAD v4.1.0 (https://gnomad.broadinstitute.org/; accessed on 16 May 2025) and 60KJPN (https://jmorp.megabank.tohoku.ac.jp/; accessed on 16 May 2025) [11], and was predicted to be pathogenic by multiple in silico prediction tools (Supplementary Table 1). The patients also possessed the c.633+1G>A variant of the *PDE6C* gene, which is known to cause inherited retinal dystrophy in an autosomal recessive manner [12]. However, since only a single allele of this variant was found in the patient’s DNA, and the patient’s phenotype was inconsistent with that typically associated with *PDE6C* variants, it was considered unlikely to be causative in our patient’s case.

**Fig. 3:**
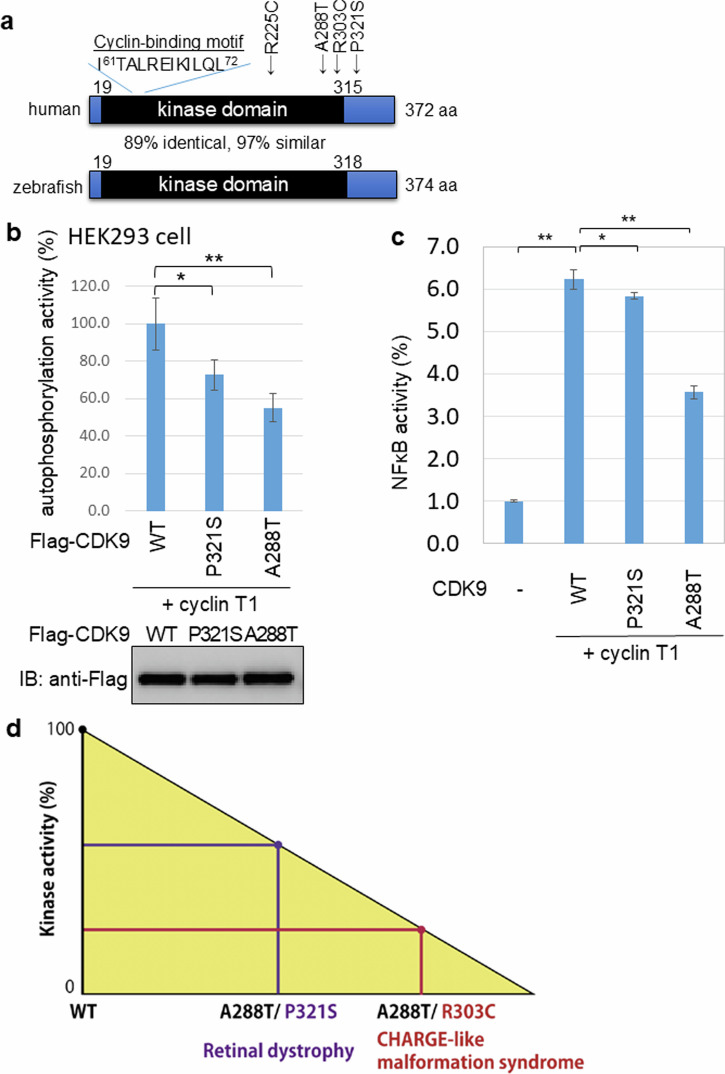
Biochemical properties of the novel CDK9 variant. **a** Diagram of the domain structure of the human and zebrafish CDK9 proteins. Numbers indicate amino acid positions from the N-terminus of human CDK9. Arrows indicate arginine (R) 225 substituted with cysteine (C), alanine (A) 288 substituted with threonine (T), arginine (R) 303 substituted with cysteine (C), and proline (P) 321 substituted with serine (S) (new variant in the patient). **b** Top: In vitro autophosphorylation assay of kinase activities of Flag-tagged human WT, P321S, and A288T CDK9 enzymes in the presence of cyclin T1. Data are the mean enzymatic activity rate ± SD of results obtained from one representative experiment in which each transfection was performed in triplicate. *p < 0.05, **p < 0.01 by Student’s *t* test. Bottom: Immunoblotting with anti-Flag antibodies to detect Flag-CDK9 proteins in the assay in the top panel. **c** NF-κB-dependent gene activity assay of HEK293 cells that were transfected with the NF-κB-Luc reporter plasmid and either Flag-tagged human CDK9 WT, P321S or A288T expression plasmids, plus CyclinT1, as indicated. After 24 h incubation, cells were harvested and luciferase activity was measured. Data are the mean values shown are the mean NF-κB activity ± SD of results obtained from one representative experiment in which each transfection was performed in triplicate. *p < 0.05, **p < 0.01 by Student’s *t* test. **d** Diagram of the proposed impact of pathological human *CDK9* biallelic variants. Assuming that the kinase activity of wild-type CDK9 is 100%, the total kinase activities of the biallelic CDK9 variants A288T/P321S and A288T/R303C are about 60% and 30% of the WT value, respectively. A patient bearing A288T/P321S would show only retinal dystrophy, whereas a patient with A288T/R303C would exhibit both retinal dystrophy and CHARGE-like malformation syndrome

As previously determined, the orthologous *CDK9* genes in human and zebrafish are highly conserved in structure and amino acid sequence, and show an identical cyclin-binding motif (Fig. 3a). The amino acid residue A288 is located in the protein kinase catalytic domain of *CDK9*, but the P321S residue mutated in our patient lies outside this region in both species. To compare the kinase activities of the P321S and A288T variants, we prepared Flag-tagged recombinant versions of the wild-type (WT), P321S and A288T CDK9 proteins and expressed them in HEK293 cells. Interestingly, the kinase activities of both the P321S and A288T variants were reduced compared to the WT enzyme (Fig. 3b). To test the relative effects of these lower CDK9 kinase activities on gene expression patterns, we employed a NF-κB reporter gene assay. We observed that the levels of NF-κB activity induced by the three CDK9 proteins could be ranked as WT 100% > P321S 70% > A288T 50% (Fig. 3c). These results clearly show that the patient’s novel P321S variant impairs CDK9 kinase activity but not to the same extent as the A288T variant, which may result in a milder phenotype (Fig. 3d). Both variants were classified as variants of uncertain significance (VUS) according to the ACMG/AMP guidelines [13] refined by the ClinGen Sequence Variant Interpretation (SVI) Working Group [14–16] (Supplementary Table 1).

## Discussion

In this study, we report that a novel compound heterozygous combination of *CDK9* missense variants [p.(A288T/P321S)] are associated with human eye disease in the absence of a multiple malformation syndrome. Previously, we showed that a patient who was compound heterozygous for a different combination of *CDK9* variants [p.(A288T/R303C)] did exhibit a multiple malformation syndrome resembling CHARGE syndrome, a classic disorder featuring multiple malformations and caused by *CHD7* variant [6]. Notably, the ocular phenotype of retinal dystrophy seen in patients bearing *CDK9* variants has yet to be associated with CHARGE syndrome [17], leading to their diagnosis as having “CHARGE-like malformation syndrome”. In this study, we identify a patient who is a compound heterozygote for two different *CDK9* variants but who does not display CHARGE-like malformation syndrome, which therefore may extend phenotypic spectrum of biallelic *CDK9* variants. Since the two variants in *CDK9* in this case were currently classified as VUS, there remains the possibility that these variants are actually irrelevant to the eye disorder. However, it is often difficult to classify missense variants as pathogenic or likely pathogenic for rare genes such as *CDK9* with limited reported cases, and accumulation of further cases will be essential.

At the molecular level, CDK9 phosphorylates RNA polymerase II and regulates transcription, whereas CHD7 has been implicated in nucleosome remodeling during transcriptional regulation. Both CDK9 and CHD7 share the same transcriptional control, but their downstream targets differ. Therefore, both similarities and differences in the phenotypes of patients bearing *CDK9* vs. *CHD7* variants are expected to occur. Differentiating CHARGE syndrome from CHARGE-like syndrome is important for genetic counseling because of their different inheritance modes. CHARGE syndrome is an autosomal dominant condition, and thus the risk for recurrence in subsequent offspring will be low when the parents are unaffected. In contrast, the *CDK9*-related CHARGE-like syndrome is an autosomal recessive condition and thus carries a high recurrence risk (25%) for these parents.

In considering phenotypic intensity from the standpoint of loss of CDK9 kinase activity, the known variants can be ranked WT > P321S > A288T > R303C, where the R303C variant (also in the kinase domain) results in the most debilitating abnormalities [6]. Accordingly, the intensity of disease in patients with p.(R303C/A288T) will be far greater than in patients with p.(P321S/A288T) (Fig. 3d). While the A288T and R303C variants both directly affect the kinase domain, P321C is outside this region and so its effect on kinase activity appears to be limited.

We consider the retinal dystrophy observed in our previous case and in the present case to be an essential phenotype associated with *CDK9* variants. Both cases developed retinal dystrophy in the fundus causing night blindness and photophobia around the age of 8. It was diagnosed by ERGs and OCT and resulted in progressive visual impairment. One previous 11-year-old patient with R303C variants also was reported to have retinal dystrophy [4]. However, the other four patients with R303C variants were not diagnosed with retinal dystrophy, probably because they were too young to have developed the disease, or because it was difficult to examine the fundus due to congenital cataracts and microphthalmia and ERGs could not be performed [3, 4]. We speculate that, depending on the impact on CDK9 kinase activity of a given *CDK9* variant, retinal dystrophy with or without CHARGE-like malformation syndrome may occur.

## Materials and methods

Materials and methods for Fig. 3b, c.

### Cell culture and transfection

Human embryonic kidney 293 (HEK293) cells were cultured in Dulbecco’s modified Eagle’s medium (DMEM) supplemented with 10% fetal bovine serum (FBS). Polyethylenimine MW 25000 (Polysciences, Warrington, PA) were used for transfection.

### Autophosphorylation assays for CDK9 variants

HEK293 cells (1.8 ×106 cells/dish) were seeded into 100 mm dishes and cultured for 24 h. These HEK293 cells were then transfected with 8 ug of expression plasmid encoding Flag-tagged WT CDK9 protein or its CDK9 P321S or CDK9 A288T variants, plus 4 ug of the Cyclin T1 expression plasmid. After 24 h to allow expression, each fusion protein was immunoprecipitated with anti-Flag antibody (Sigma, M2 agarose). Immunoprecipitates were purified by washing six times with PBS. The kinase activity of the CDK9 variants under study was evaluated using an in vitro autophosphorylation assay as previously described [18]. **A**liquots of immunoprecipitated Flag-tagged WT or CDK9 variant proteins were incubated at 25 °C for 90 min with or without 50 μM ATP in 40 μL of kinase buffer containing 40 mM Tris (pH7.5), 100 μM BSA, and 20 mM MgCl_2_. The ADP produced by the autophosphorylation reaction was measured using the ADP-Glo® Kinase Assay kit (Promega). Immunoprecipitated proteins from cells transfected with empty vector were used to measure background activity.

### NF-κB reporter gene assays for CDK9 variants

HEK293 cells (3 × 10^5^ cells/well) were seeded into six-well (35 mm) plates and cultured for 24 h. Cells were transfected as above at 24 h after seeding with 0.5 μg NF-κB-Luc reporter gene plasmid (Ig-κ-luciferase reporter) plus 1 ng pRL-EF vector, along with 0.25 μg of either Flag-tagged WT CDK9, CDK9 P321S or CDK9 A288T and CyclinT1 expression vector. The pRL-EF plasmid expressing *Renilla* luciferase under the control of the EF-1α promoter was used to normalize transfection efficiency of the luciferase reporters. The total concentration of transfected DNA (2 μg) was kept constant by supplementing with empty vector DNA. After culture of transfected cells for 48 h, Firefly and *Renilla* luciferase activities were determined using the Promega Dual luciferase assay system.

## Supplementary information

Supplementary Table 1
