# Supplementary material for: Novel biallelic CDK9 variants are associated with retinal dystrophy without CHARGE-like malformation syndrome
Source: J Hum Genet. 2025 Sep 16;71(2):97–103. doi: 10.1038/s10038-025-01395-1 (PMC12765697; doi:10.1038/s10038-025-01395-1)
Supplement: Supplementary file 1 — Supplementary Table 1 [file 10038_2025_1395_MOESM1_ESM.pdf]

Supplementary Table 1. Detected *CDK9* variants with allele frequencies, *in silico* prediction scores, and conservation scores

Identified variants					Allele frequencies		Functional prediction scores							Evolutionary conservation scores		ACMG/AMP criteria	
Gene (cytoband)	Accession number	Nucleotide change	Amino acid change	Inheritance	60KJPN	gnomAD v4 (global)	CADD	polyphen-2	SIFT	M-CAP	REVEL	Alpha Missense	GERP++RS	phyloP100way	Applied evidence	Classification	
CDK9 (9q34.11)	NM_001261.4	c.862G>A	p.A288T	matermal	0.00134	0.00002293	27.7	1	0	0.133999	0.715	0.931	5.43	9.702	PS3_supporting, PM2_supporting, PP3	VUS	
		c.961C>T	p.P321S	de novo	0	0	25.3	1	0	0.059809	0.397	0.993	5.36	7.67	PS2_moderate, PS3_supporting, PM2_supporting	VUS	

60KJPN: Allele frequency data of SNV/INDEL from short-read whole genome sequencing of 60,000 Japanese individuals (https://jmorp.megabank.tohoku.ac.jp/)

gnomAD v4.1.0 (https://gnomad.broadinstitute.org/)

CADD (http://cadd.gs.washington.edu/): Scores > 20 indicate that the variant is among the top 1% most deleterious possible substitutions in the human genome.

PolyPhen-2 (http://genetics.bwh.harvard.edu/pph2/): Scores range from 0.000 (benign) to 1.000 (probably damaging).

SIFT (https://sift.bii.a-star.edu.sg/): Scores ≤ 0.05 are considered damaging; scores > 0.05 are considered tolerated.

M-CAP (https://bejerano.stanford.edu/mcap/): Scores > 0.025 suggest the variant is likely pathogenic.

REVEL (https://sites.google.com/site/revelgenomics/): Scores range from 0 to 1; higher scores indicate greater likelihood of pathogenicity.

AlphaMissense (https://alphamissense.hegelab.org/): Scores range from 0 to 1; higher scores indicate greater predicted pathogenicity.

GERP++_RS: Scores > 2.0 suggest evolutionary constraint.

phyloP100way: Positive scores indicate conservation; negative scores suggest acceleration.
